# Foreign Body Reaction Associated with PET and PET/Chitosan Electrospun Nanofibrous Abdominal Meshes

**DOI:** 10.1371/journal.pone.0095293

**Published:** 2014-04-16

**Authors:** Beatriz Veleirinho, Daniela S. Coelho, Paulo F. Dias, Marcelo Maraschin, Rúbia Pinto, Eduardo Cargnin-Ferreira, Ana Peixoto, José A. Souza, Rosa M. Ribeiro-do-Valle, José A. Lopes-da-Silva

**Affiliations:** 1 QOPNA Research Unit, Department of Chemistry, University of Aveiro, Aveiro, Portugal; 2 Biotechnology and Biosciences Post-Graduation Program, Federal University of Santa Catarina, Florianópolis, Brazil; 3 Department of Cell Biology, Embryology, and Genetics, Federal University of Santa Catarina, Florianópolis, Brazil; 4 Plant Morphogenesis and Biochemistry Laboratory, Federal University of Santa Catarina, Florianópolis, Brazil; 5 Epagri, Florianopolis, Brazil; 6 Federal Institute of Education, Science, and Technology of Santa Catarina, Garopaba, Brazil; 7 Department of Pediatrics, Federal University of Santa Catarina, Florianópolis, Brazil; Instituto de Engenharia Biomédica, University of Porto, Portugal

## Abstract

Electrospun materials have been widely explored for biomedical applications because of their advantageous characteristics, i.e., tridimensional nanofibrous structure with high surface-to-volume ratio, high porosity, and pore interconnectivity. Furthermore, considering the similarities between the nanofiber networks and the extracellular matrix (ECM), as well as the accepted role of changes in ECM for hernia repair, electrospun polymer fiber assemblies have emerged as potential materials for incisional hernia repair. In this work, we describe the application of electrospun non-absorbable mats based on poly(ethylene terephthalate) (PET) in the repair of abdominal defects, comparing the performance of these meshes with that of a commercial polypropylene mesh and a multifilament PET mesh. PET and PET/chitosan electrospun meshes revealed good performance during incisional hernia surgery, post-operative period, and no evidence of intestinal adhesion was found. The electrospun meshes were flexible with high suture retention, showing tensile strengths of 3 MPa and breaking strains of 8–33%. Nevertheless, a significant foreign body reaction (FBR) was observed in animals treated with the nanofibrous materials. Animals implanted with PET and PET/chitosan electrospun meshes (fiber diameter of 0.71±0.28 µm and 3.01±0.72 µm, respectively) showed, respectively, foreign body granuloma formation, averaging 4.2-fold and 7.4-fold greater than the control commercial mesh group (Marlex). Many foreign body giant cells (FBGC) involving nanofiber pieces were also found in the PET and PET/chitosan groups (11.9 and 19.3 times more FBGC than control, respectively). In contrast, no important FBR was observed for PET microfibers (fiber diameter = 18.9±0.21 µm). Therefore, we suggest that the reduced dimension and the high surface-to-volume ratio of the electrospun fibers caused the FBR reaction, pointing out the need for further studies to elucidate the mechanisms underlying interactions between cells/tissues and nanofibrous materials in order to gain a better understanding of the implantation risks associated with nanostructured biomaterials.

## Introduction

Electrospinning has attracted the interest of researchers from many fields as a versatile technique to produce nanofibers from synthetic and naturally derived polymers. With typical diameters ranging from 10 nm to a few micrometers, these fibers are usually collected continuously as nonwoven fibrous mats. These mats usually show a tridimensional nanostructure with high surface-to-volume ratio, high porosity, and interconnectivity, and they have demonstrated high potential for biomedical applications, such as tissue engineering scaffolds, vascular grafts, and drug delivery systems [1]–[3].

In the past decade, our research group has been exploring the potential of electrospinning for different applications [4]. Cell culture studies revealed that hybrid nanofibers of poly(ethylene terephthalate) (PET) and chitosan provide a good substratum for fibroblast adhesion, proliferation, extracellular matrix secretion, and three-dimensional colonization [5], in addition to their interesting surface and mechanical properties [6], [7]. The promising results obtained *in vitro* prompted us to test these nondegradable electrospun mats as abdominal meshes for incisional hernia repair.

Incisional hernia is a frequent complication of laparotomy resulting from the decrease of abdominal strength in the injured tissue. The incidence of incisional hernia after abdominal surgery depends on the pattern of the incision performed. For a midline incision, the preferred incision for the upper abdominal surgery, the incidence lies around 10–14% [8]–[10]. Different patterns of incision, however, such as the transverse incision, yield much lower rates of hernia formation (2%) [10]. In comparison to the traditional hernia repair strategy by primary closure, the implementation of a tension-free repair by using a prosthetic biomaterial, i.e., nonresorbable abdominal mesh, to substitute or reinforce abdominal strength at the damaged area has decreased the recurrence rates significantly [8]–[12]. Nevertheless, serious complications have also been associated with this procedure, including infection, visceral adhesions to the mesh, seroma, mechanical failure of the mesh, and foreign body reaction [13], [14]. Bowel adhesion to the implanted mesh is a major concern as it causes serious complications, including bowel obstruction, enterocutaneous fistula, and chronic pain [15].

Absorbable meshes are also used as scaffolds for abdominal defect correction. In this case, the biodegradable mesh provides support for cell growth and for extracellular matrix secretion, promoting the tissue repair process. A few recent reports have shown successful results in exploring partially degradable or absorbable electrospun mats as abdominal meshes. An electrospun blended fiber mesh prepared from biodegradable poly(ester urethane) urea and poly(lactide-*co*-glycolide), latter loaded with an antibiotic, was shown to provide good mechanical properties, while imparting antibacterial activity and, hence, reducing the risk of infection during application of the composite material to abdominal wall closure [16]. A similar approach used electrospun poly(ester urethane) urea fibers deposited with electrosprayed serum-based culture medium [17] or porcine dermal extracellular matrix digest [18]. When these materials were tested as abdominal wall repair meshes, they were demonstrated to provide adequate mechanical properties and, at the same time, enhanced bioactivity, biocompatibility and cell infiltration, with no herniation, infection, or tissue adhesion. Other examples include the application of electrospun absorbable polycaprolactone scaffolds [19], which were also evaluated for their suitability in hernia repair.

Despite the advantages of absorbable materials, the newly formed tissue has a decreased tensile strength and thereby, re-herniation is a frequent problem after the absorption of the prosthetic material. In this context, electrospun mats of nondegradable polymers have emerged as potential alternative meshes for abdominal defect repair. Because of its unique properties, i.e., unaligned nanofibrous arrangement, microporosity, and high hydrophobicity, the PET electrospun mat has emerged as a potential candidate for abdominal wall repair [6]. In addition to these advantages, PET is a highly biocompatible, biostable, and nondegradable polymer which possesses the mechanical features required for this application. Moreover, the low density (∼ 0.091 g/cm^−3^) and the high malleability of this material may promote enhanced adaptation and, consequently, patient comfort [7]. On the other hand, as a hydrophobic material with small pores, electrospun PET meshes may restrict the integration of parietal conjunctive tissue. McGinty *et al.* have demonstrated that a better incorporation of the mesh in the parietal side reduces the number and the severity of adhesion formations on the visceral side [20]. Hence, with the aim of enhancing the interaction of the mesh with the parietal conjunctive tissue, a hybrid mat of PET/chitosan (PET/C) was developed. Chitosan has shown ideal properties for biomedical applications, including the anti-inflammatory and wound healing effects, which may attenuate the typical symptoms of the post-surgery period and prevent adhesiogenesis [21], [22]. Additionally, in the current study, a double-layered mesh (DL), containing one layer of PET (turned to the visceral side) and one layer of PET/C mat (turned to the parietal side), was also developed.

In this paper, we describe the application of three electrospun nonabsorbable mats, including PET, PET/C and DL, in the repair of abdominal defects, comparing the performance of these nanofibrous meshes with that of a commercial polypropylene mesh (Marlex) (control) and a multifilament microfibrous PET mesh. An *in vivo* study with an abdominal hernia Wistar rat model was performed to evaluate the clinical and histological aspects of using these meshes for abdominal hernia repair.

## Materials and Methods

Animal experiments were approved by the Animal Ethics Committee of the Federal University of Santa Catarina (PP0406/2009).

### Materials

PET pellets and PET woven fabric were kindly supplied by Flexitex (Portugal). Marlex (Intracorp) was purchased from *Cirurgica Passos,* Brazil. Chitosan medium molecular weight (15% acetylation degree) was purchased from Sigma-Aldrich Chemical Company. The molecular weight of the initial chitosan sample (1500 kDa) was reduced to 15 kDa by oxidative depolymerization [23]. All chemicals were of analytical grade and obtained from Sigma-Aldrich Chemical Company.

### Mesh Fabrication by Electrospinning

Thirty percent (w/v) PET solution was prepared in a blend of trifluoroacetic acid and dichloromethane [8∶2 v/v] by moderate stirring for 2 hours at room temperature. PET/C blend was prepared by adding chitosan (6 wt. %) to the PET 30 wt. % solution and stirring for 3 hours at room conditions.

Electrospinning was performed using a typical experimental setup previously described [6]. The process was conducted at 26 kV of applied voltage, with a flow rate of 0.08 mL/min (V = 20 mL) and a needle tip-to-collector distance of 12 cm.

The double-layer (DL) mesh was fabricated by electrospinning 10 mL of PET solution, followed by 10 mL of PET/C solution. Fibers were collected as a nonwoven fibrous mat on the rotating drum (900 rpm), in air and at room conditions (20±2°C, 45–50% RH), and the mat was dried at 35°C for 24 hours.

### Morphological Analysis

The morphology of the fibrous scaffolds was investigated by scanning electron microscopy (SEM). Small sections of the scaffolds were sputter-coated with gold and analyzed using a scanning electron microscope (Hitachi S4100) at an accelerating voltage of 25 kV.

Image processing was performed using ImageJ - 1.37c software (Wayne Rasband, National Institutes of Health, USA). Five random images (1000 X magnification) were obtained for each sample. Fiber diameters were calculated from at least 100 measurements of the sample fibers. SEM images were thresholded, and pore areas were automatically calculated using the “analyze particle” tool of ImageJ (n >200).

### Mechanical Properties

Mechanical properties in tension were evaluated using texture analyzer equipment (Model TA HDi, Stable Micro Systems, England) equipped with fixed grips lined with thin rubber on the ends. Test specimens 90 mm long×10 mm wide were obtained perpendicular to the axis of the collector rotation, and the ends were mounted on the grips using sticky tape. The thickness of the test samples was measured at different locations on each sample using a digital micrometer (Model MDC-25S, Mitutoya Corp., Tokyo, Japan). The initial grip separation was set at 50 mm, and the crosshead speed was 0.5 mm/s. At least eight samples of each mat were tested.

### Water Contact Angle (WCA) Measurements

The wettability of scaffolds was assessed by the sessile drop method using an OCA-20 contact angle system (DataPhysics Instruments). A drop of distilled water (1 µL) was automatically dispensed on the scaffold surface, and the WCA and drop life times were calculated using the SCA 20 software. At least 10 measurements were taken for each sample.

### Animal Model

Male Wistar rats 3 months of age and weighing 250–300 g were obtained from the Central Biotery of the Federal University of Santa Catarina. Animals were randomly distributed among the 7 treatments (Marlex30, PET30, PET/C, DL, Woven-PET, Marlex90, and PET90), according to Table 1 (n≥8).

**Table 1:** Experimental groups of incisional hernia repair.

Group	Mesh chemical composition	Duration of experiment (days)	Mesh thickness (mm)
Marlex30	Polypropylene	30	0.22±0.07
Marlex90	Polypropylene	90	0.22±0.07
PET30	PET	30	0.31±0.02
PET90	PET	90	0.31±0.02
Woven-PET	PET	30	0.49±0.09
PET/C	PET/C 5∶1 (w/w)	30	0.52±0.05
DL	PET + PET/C 5∶1 (w/w)	30	0.46±0.11

Before biological assay, all meshes were sterilized under UV light for 1 hour of exposure (both faces), immersed in ethanol 70% (v/v) for 10 minutes, and washed with sterile physiologic solution.

A graphical illustration of the surgical procedure is provided (Figure S1). After an intramuscular injection of a mixture of ketamine (90 mg/kg) and xylazine (15 mg/kg) and abdominal shaving, a 5 cm paramedian skin incision was made at the left side, using a sterile scalpel blade. Skin was dissected to expose the underlying abdominal fascia, and a 1.5×1.5 cm defect of anterior abdominal wall was created by the complete resection of abdominal layers. The edges of the meshes (2.0×2.0 cm) were sutured to the remaining muscle of the abdominal wall with interrupted suture and also with simple running suture all over the borders, using 5–0 polypropylene. The skin was closed with intradermal suture with nylon 4–0 monofilament. Animals were allowed to recover from anesthesia, housed in individual cages, and observed daily for evidence of wound complications, such as redness, infection, seroma, abcess, hematoma, or skin dehiscence.

On day 30 and 90 post-surgery, animals were sacrificed in a carbon dioxide chamber, and the presence of intestinal adhesions was analyzed. The abdominal wall was carefully excised well away from the mesh (see Figure S2) to preserve any adherence to the bowel or omentum. After adhesion analysis, tissue was completely excised and collected for histopathological analysis.

### Histopathological Analysis

Tissues were excised from the animals and fixed in phosphate buffered formaldehyde solution (4%, pH 7.2, 0.1 M), embedded in paraffin, and sectioned at 4 µm thickness. Giemsa, hematoxylin and eosin (HE), and Garvey’s staining were performed [24]. For immunohistochemical analysis, sections were deparaffinized in xylene and rehydrated in a graded ethanol series. Antigen retrieval was performed with 0.05% trypsin and 0.1% calcium chloride (20 minutes, 37°C). Endogenous peroxidase activity was blocked by incubation in a hydrogen peroxide solution. Following, sections were incubated with a monoclonal antibody directed against CD68, clone KP1, dilution 1∶100 (Zeta Corporation, CA). Antibody detection was performed using Histofine Simple Stain Max-Po Multi (Nichirei Biosciences, Tokyo, Japan) and 3,3′-diaminobenzidine tetrahydrochloride (Spring Bioscience, CA). Samples were analyzed under a microscope (Nikon Eclipse 50i equipped with a Nikon Digital Sight DS-Fi2). The thickness of the foreign body granuloma was measured and the absolute number of foreign body giant cells (FBGC) per granuloma section was determined by manual counting (n≥8). A detailed description of morphometric procedures is given in Figure S2.

### Statistics

Statistical analysis was carried out using Instat 3.0 software. Results were expressed as the mean ± standard deviation and compared through one-way ANOVA and Tukey-Kramer.

## Results

### Characterization of Meshes

**Figure 1:**
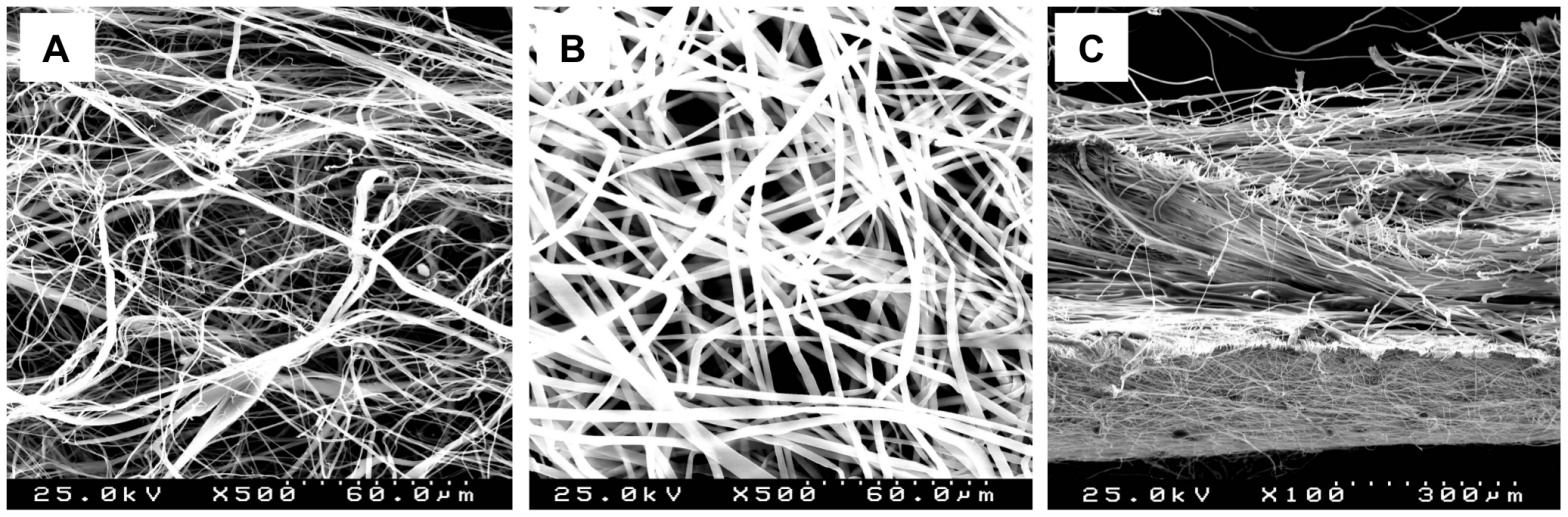
Morphological analysis of the meshes. SEM images of (A) PET, (B) PET/C, and (C) DL meshes. The transversal section of the DL mesh shows PET mat (bottom) and PET/C mat (top). *Bars*: 60 µm (A, B); 300 µm (C).

Figure 1 displays SEM images of PET, PET/C, and DL mats. The electrospun mats showed a typical nonwoven fibrous structure with random fiber orientation and high porosity. Table 2 summarizes some morphological, mechanical, and surface properties of the meshes used in this study. The average diameter of PET fibers was 0.71±0.28 µm, and the average pore area was 9.4 µm^2^. Addition of chitosan promoted a substantial increase in fiber diameter and pore area to 3.01±0.72 µm and 89.3 µm^2^, respectively. Compared to PET/C, PET mesh showed superior mechanical properties with a higher tensile strength (3.17±0.23 MPa compared to 2.89±0.27 MPa), Young’s modulus (120±10 compared to 70±10 MPa), and elongation (32.8±5.7% compared to 8.2±1.3%). Also, a decrease in the hydrophobicity of the mesh was observed by the presence of chitosan (WCA decreased from 133.2±2.9° to 125.2±4.6° with the addition of chitosan).

**Table 2:** Fiber diameter, pore area, mechanical properties, and WCA of abdominal meshes.

Group	Fiber diameter (µm)	Pore area (µm^2^)	Tensile strength (MPa)	Percentage elongation (%)	Young’s modulus (MPa)	WCA (°)
Marlex	177±24	31400	N.A.	N.A.	N.A.	N.A.
PET	0.71±0.28	9.4	3.17±0.23	32.8±5.7	1.2±0.1	133.2±2.9
PET/C	3.01±0.72	89.3	2.89±0.27	8.2±1.3	0.7±0.1	125.2±4.6
Woven-PET	18.9±2.1^a^	N.A.	N.A.	N.A.	N.A.	N.A.
	342±68^b^					

N.A. not available

a Average filament diameter.

b Average diameter of the multifilament yarn.

### Surgical Procedure and Post-operative Period

The electrospun mats were evaluated in an incisional hernia experiment with Wistar rats and compared to Marlex, the control. Following the creation of the abdominal defect by resection of 1.5×1.5 cm of the Wistar rats’ abdominal muscle, the prosthetic meshes were implanted and fixed through the borders to the remaining muscle (see Figure S1). Suture of electrospun meshes was easily performed without breakage. In fact, electrospun meshes were more suitable and more resistant to suture than control, where an extra margin of around 5 mm was used to avoid breaking of filaments.

During the post-operative period, some complications, such as local redness, abscess, or skin dehiscence, were registered. Table 3 displays macroscopic complications found in the post-operative period. No complications were found during the post-operative period for the woven-PET group. In the other experimental groups, local redness, characterized by redness or swelling, was the most common complication, with higher incidence of these complications observed for PET/C and DL groups, occurring in 50% and 75% of the animals, respectively. Redness tended to decrease with time, while abscess and skin dehiscence persisted until the end of the experimental period. Omentum adhesions to the mesh were observed in all animals (see Figure S3), but no visceral adhesions were found in the experimental groups.

**Table 3:** Occurrence rate (%) of complications during post-operative period (PO) and day euthanized (E).

Mesh	Period	Local redness	Dehiscence	Seroma or abscess	Adhesion
**Marlex**	PO	37.5	12.5	12.5	N.A.
	E	12.5	12.5	12.5	omentum
**PET**	PO	37.5	0	12.5	N.A.
	E	25	0	12.5	omentum
**PET/C**	PO	50	0	0	N.A.
	E	0	0	0	omentum
**DL**	PO	75	12.5	12.5	N.A.
	E	37.5	12.5	12.5	omentum
**Woven- PET**	PO	0	0	0	N.A.
	E	0	0	0	omentum

N.A. not available.

### Histological Analysis

**Figure 2:**
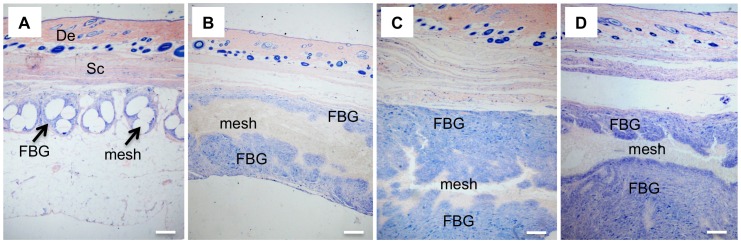
Foreign body granuloma induced by the abdominal meshes. Histological sections evidencing foreign body granuloma (Giemsa staining) of (A) Marlex30, (B) PET30, (C) PET/C, and (D) DL groups. (De) dermis, (Sc) subcutaneous tissue, and (FBG) foreign body granuloma. Bar = 220 µm.

**Figure 3:**
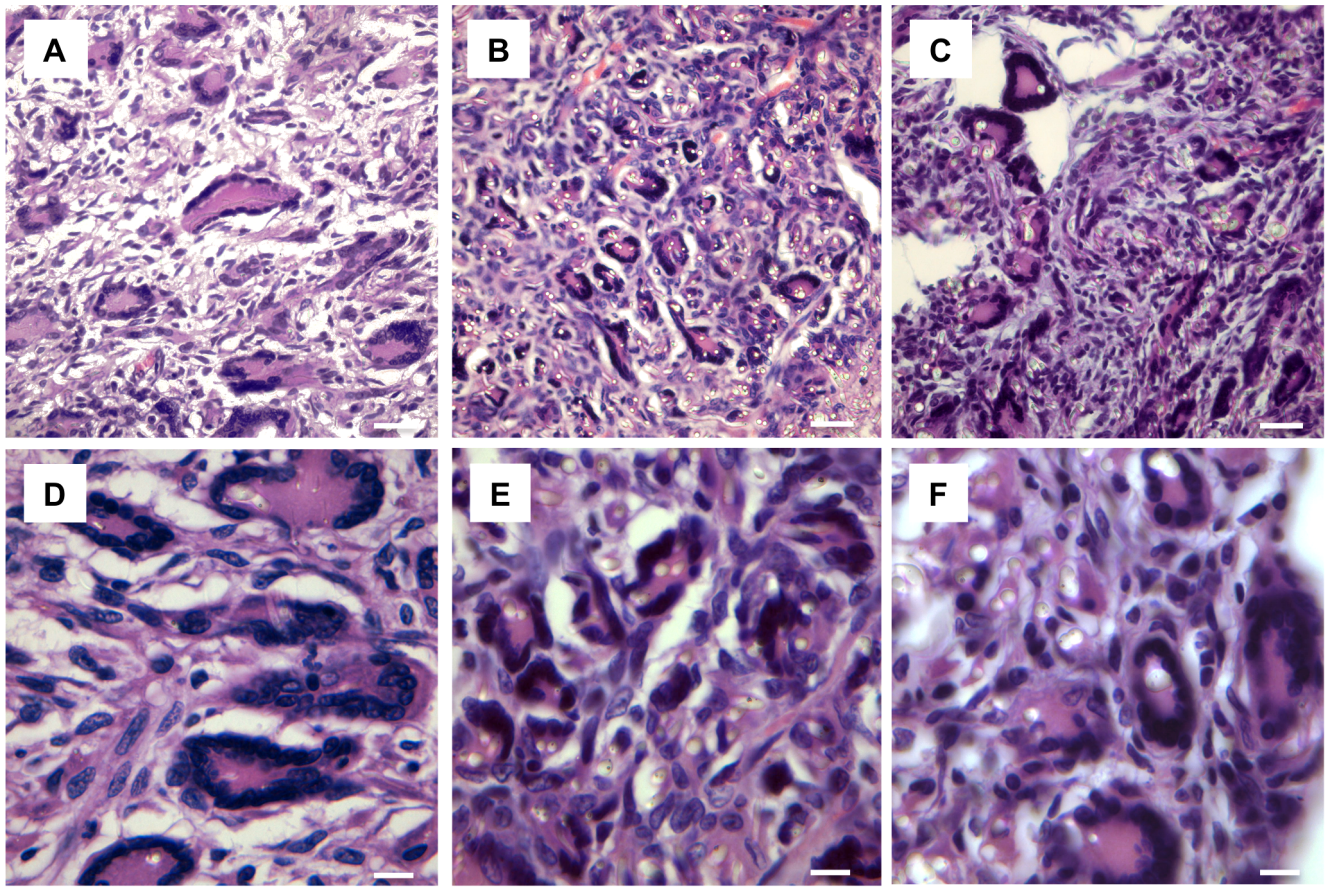
Foreign body giant cells covering nano- and microfibers of electrospun materials. Histological sections (HE staining) evidencing FBGC in (A and D) PET30, (B and E) PET/C, and (C and F) DL groups. *Bars*: 25 µm (A–C); 10 µm (D–F).

**Figure 4:**
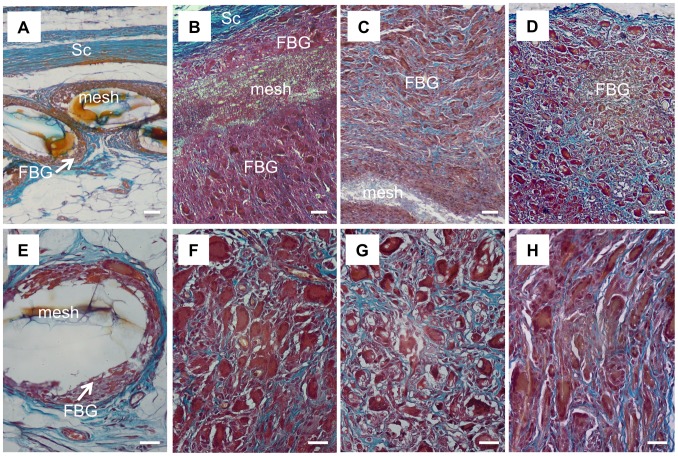
Garvey staining of foreign body granulomas. Histological sections stained with Garvey’s staining (nuclei stained black/dark red, cytoplasmic elements stained red and collagen fibers stained light blue). (A,E) Marlex (B,F) PET (C,G) PET/C (D,H) DL. (De) dermis, (Sc) subcutaneous tissue, and (FBG) foreign body granuloma. Bars = 50 µm (A–D); 25 µm (E–H).

**Figure 5:**
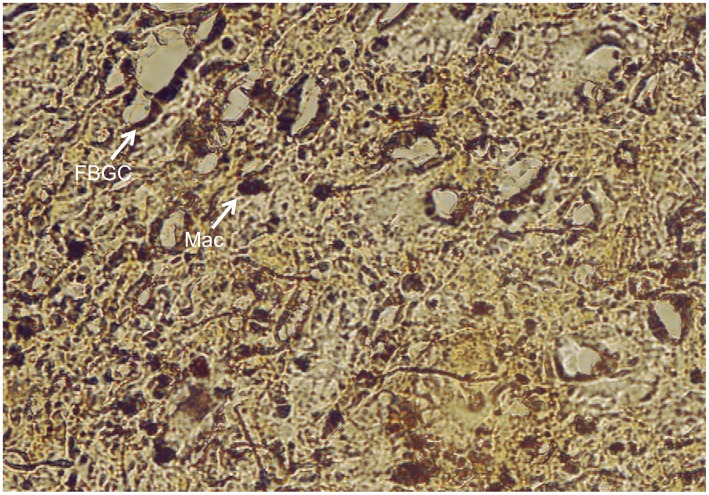
Immunohistochemistry for CD68. Immunohistochemical staining (CD68+) evidencing macrophages (Mac) and foreign body giant cells (FBGC) around a PET electrospun mat implanted in rat as abdominal mesh. Sections were incubated with a monoclonal antibody directed against CD68, clone KP1 (Zeta Corporation, CA), Histofine Simple Stain Max-Po Multi and 3,3′-diaminobenzidine tetrahydrochloride.

**Figure 6:**
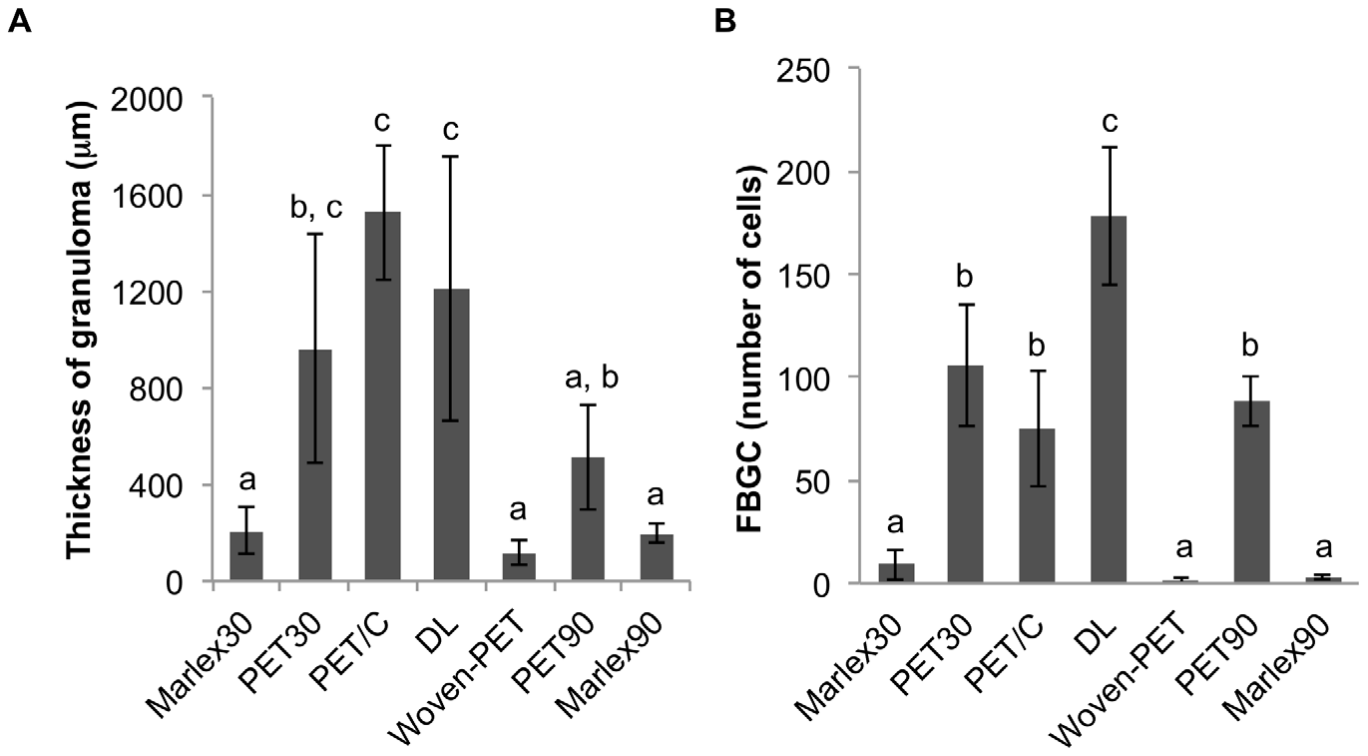
Analysis of the foreign body granuloma induced by abdominal meshes. (A) Granuloma thickness (mean ± standard deviation) and (B) absolute number of FBGC per section of granuloma (mean ± standard deviation) in animals implanted with different abdominal meshes. All experimental groups were compared to each other using Tukey-Kramer multiple comparison tests. For each chart, bars with different letters are significantly different at P<0.05.

Histological analysis was performed to evaluate the cellular response to the prosthetic biomaterials. Figure 2 and Figure 3 display representative images of HE-stained sections of animals treated with electrospun meshes and Marlex (low and high magnification, respectively). All animals showed typical nonimmunogenic granulomas (foreign body granulomas) surrounding the mesh structure placed below the abdominal subcutaneous tissue. Representative photomicrographs from Garvey’s staining are provided in Figure 4. The foreign body granuloma were mostly composed of macrophages, foreign body giant cells (FBGC), and fibroblasts. Multinucleated cells frequently involved one or more fiber segments. Immunohistochemical analysis confirmed the high density of both macrophages and FBGC (CD68+) in animals treated with the electrospun materials (Figure 5). The mean thickness of the granuloma and the average number of FBGC in the granuloma are plotted in Figure 6A and Figure 6B, respectively. Animals treated with electrospun meshes showed significantly thicker granulomas and a higher number of FBGC compared to Marlex and the woven-PET group. The mean granuloma thickness induced by PET nanofibers was 4-fold higher than control (Marlex) and 10-fold higher than in the woven-PET group. Also a 10-fold increase in the number of FBGC was observed in the PET group compared to control. Hybrid meshes showed even thicker granulomas (1522±277 µm and 1211±547 µm for PET/C and DL, respectively), as well as a large number of FBGC comprising one or more fibrous structures. The woven-PET group produced the weakest inflammatory response with an average granuloma thickness of 87±35 µm and FBGC rarely observed.

Long-term inflammatory response was evaluated for two selected groups (Marlex and electrospun PET) 90 days after mesh implantation. Both groups showed a decrease in inflammation with time. The average granuloma thickness of the electrospun PET group decreased from 959±473 µm to 513±217 µm, and the number of FBGC decreased from 106±30 to 89±12.

## Discussion

Several biomaterials have been used as prosthetic meshes for abdominal wall repair, in particular, incisional hernia repair. Among them, nonabsorbable polymers with recognized biocompatibility, such as polypropylene, PET or polytetrafluoroethylene, are the most common. Although a considerable improvement in the recurrence rate has been achieved with these materials in comparison to the traditional suture technique, several problems are still associated with this procedure. Among them, the formation of adhesions between the mesh and the bowel results in several complications, such as chronic pain, bowel obstruction or enterocutaneous fistula. Also, nonresorbable meshes have been associated with a high occurrence of chronic foreign body response and increased risk of infection [25], [26]. Improvements have been achieved by using polymer meshes that gradually degrade *in vivo*, promoting improved tissue integration and rapid resorption [19]. However, as scaffolds to support native tissue ingrowth, resorbable synthetic meshes are still limited by their loss of strength. Considering the attractive properties of electrospun polymer meshes, we have developed, for the first time, electrospun mats of nondegradable polymers for the repair of abdominal defects.

Chemical composition, weight, pore size, and filament structure, represent critical parameters employed in surgical mesh design [27]. Apart from preventing adhesion, it is generally accepted that the ideal abdominal mesh should be chemically inert and stable for long periods, promote tissue regeneration to form an adequate barrier against protrusion, cause no immune or inflammatory response, and fulfill the required mechanical needs for the application. The studied PET electrospun mats are nonwoven meshes of nanofibers (average diameter = 0.71±0.28 µm) thought to be ideal for the prevention of visceral adhesion by their highly hydrophobic microporous structure (WCA = 133.2±2.9°; average pore area = 9.4 µm^2^). Indeed, according to Mathews *et al.*, biomaterials with pores smaller than 75 µm reduce the occurrence of bowel adhesion [28].

With the purpose of manipulating the architecture of PET mesh and also taking advantage of the anti-inflammatory and wound healing effects of chitosan, a hybrid fibrous mat (PET/C) was also developed, with higher fiber diameter and pore area. These morphological differences can have significant effects on cell-biomaterial interactions, as previously demonstrated [5]. The decreased stiffness of the hybrid mesh in comparison to the PET mesh may be attributed to a heterogeneous polymer distribution within fibers as a result of phase separation during the electrospinning process [7]. The increased hydrophilic character observed for the PET/C hybrid may be advantageous for mesh integration on the parietal side. A double-layer mesh was also developed, comprising one layer of PET to prevent formation of bowel adhesion and one layer of PET/C nanofibers to stimulate integration of the mesh in the subcutaneous tissue, thus reinforcing the mechanical strength of the prosthetic wall.

In the rat abdominal hernia model, electrospun meshes were demonstrated to be adequate for the surgical procedure, i.e., easy to suture, and as a soft and flexible material, the electrospun meshes adapted well to the abdominal tissues. In contrast, the stiffness of the Marlex material may affect surrounding tissues and cause discomfort. This material was even perceived through the animal’s skin by touching. Electrospun meshes, on the other hand, were much softer, as well as more malleable and adaptable, while effectively performing their role of containing visceral components without mesh failure during the experimental period.

In contrast to bowel adhesion that can lead to serious complications, the observed omentum adhesion to the meshes is considered clinically irrelevant [29]. In fact, some authors have even suggested that the interposition of omentum between the prosthetic mesh and viscera is effective in restricting omentum adhesion, both in preclinical and clinical studies [29], [30].

Importantly, we observed a significant foreign body reaction associated with the electrospun nanofibrous meshes. This was an unexpected result since the chemical composition of the electrospun mesh is 100% PET, a recognized biocompatible polymer, and many studies have reported the absence of inflammatory response to polymer electrospun fibers by different cell types, both *in vitro* and *in vivo* [31]–[34]. Numerous studies have reported on the anti-inflammatory and wound healing effects of chitosan; however, in this specific application, chitosan had a negative impact, as an increased inflammatory response was observed in animals treated with chitosan- containing meshes. Similar results were obtained by Barbosa *et al.* [35] when testing the inflammatory response to chitosan scaffolds with different acetylation degree. In fact, the chitosan scaffold with an acetylation degree of 15% induced the formation of a thick granuloma with high infiltration of inflammatory cells, after subcutaneous implantation in mice [35]. Another study also showed that chitosan a scaffold with an acetylation degree of 15% caused a macrophage M1 pro-inflammatory response [36].

The foreign body granulomas were mostly composed of macrophages, FBGC, and fibroblasts. An abundance of FBGC was found in tissues surrounding the electrospun meshes, evidencing a typical foreign body reaction. FBGC were most often found to be enclosing one or more nanofibers as an attempt to isolate the foreign material (*cf.* Figure 3). Extending the experimental time seemed to result in an attenuation of the foreign body reaction; nonetheless, a large FBR persisted 90 days after mesh implantation. Although foreign body reaction has few clinical implications and is usually limited to the close periphery of the implanted material, certain clinical disadvantages are always present as an associated risk condition. Indeed, chronic inflammation and the related proangiogenic process have been assumed to underlie most chronic diseases, including cancer, cardiovascular diseases, and diabetes [37], [38].

It is well established that polymer type, mesh construction, fiber size, mesh porosity and contact surface, as well as the specific characteristics of the tissue where the biomaterial is implanted, play important roles in biocompatibility and induced tissue reactions [39], [40]. Still, no consensus has been reached with respect to the effects of implanted mesh and the development of inflammation [41]–[43]. Indeed, cell/tissue-mesh interactions still require further elucidation.

Considering the high biocompatibility of bulk PET and the minimum foreign body reaction found for woven-PET mesh (PET-woven microfibers), we hypothesize that the nanostructure of the electrospun materials underlies the huge foreign body reaction found in animals implanted with electrospun meshes. The reduced diameter of the electrospun fibers and pore size of the meshes, combined with the high surface-to-volume ratio of the electrospun materials, may therefore have important effects on the inflammatory reaction. Among surface properties, the material’s ability to adsorb proteins plays a key role, as those proteins, not the material’s composition itself, are major contributors of FBR [40]. It is well documented that the high surface-to-volume ratios of electrospun nanofibrous materials contribute to their high protein adsorption capability [44], [45] and likely explain the high foreign body reaction observed in electrospun PET meshes. Indeed, higher foreign body reactions are often found for biomaterials, in both in micro- and nanoscale dimensions, with large surface areas [46], [47]. Specifically, in abdominal defect repair, Conze *et al.* [48] have verified a pronounced foreign body reaction for a multifilament small-diameter polypropylene mesh. In fact, the diameter of foreign body granuloma 90 days after mesh placement decreased from 106.5 µm to 70.9 µm by increasing the filament diameter from 0.6 mm to 2.5 mm. On the other hand, many reports show a decrease of inflammatory response with the decrease of fiber diameter. Saino *et al.* [49] showed that the decrease of fiber diameter of electrospun polylactic acid fibrous mats reduced *in vitro* macrophage activation and the secretion of proinflammatory molecules. Similarly, Cao *et al.* [50] demonstrated the importance of the nanofibrous scaffold architecture and topography on the *in vivo* and *in vitro* foreign body reaction and showed a decrease of granuloma thickness of subcutaneous implants from 38 µm for a PCL film to ∼8 µm and 4 µm for PCL-aligned electrospun nanofibers or nonwoven electrospun nanofibers, respectively.

Other aspects that are thought to have influenced the observed intensive foreign body reaction are related to the extension of the trauma and the specific characteristics of the tissues where the materials had been implanted. Abdominal mesh implantation involves the creation of a 1.5×1.5 cm defect by complete resection of the abdominal wall. This is a severe trauma, considering the relative size of the animal, and, consequently, a large inflammatory response may be induced. Furthermore, specific characteristics of the implantation tissues, such as cell composition and function, vascularization, extracellular matrix composition, and contact with ascitic fluid, for example, have important effects on foreign body reaction.

Materials are becoming smaller than the basic body unity, i.e. the cell. Despite the enormous progress of the electrospinning technique over the past decade, cellular response and the associated risks involved in the use of nanostructure fibrous biomaterials are still poorly understood [51], [52], and further studies are needed to gain more insight.

## Conclusions

PET and PET/chitosan electrospun meshes demonstrated good performance during the implantation surgery, adequate mechanical attributes, and no evidence of intestinal adhesion. Nevertheless, a large foreign body reaction was found in animals treated with the electrospun mats. Indeed, the reduced dimension of nanofibers and the high surface-to-volume ratio of electrospun nonwoven materials may induce a high foreign body reaction, depending on the extent and location of the lesion. Refinement may be achieved by the inclusion of biological components on the fiber’s surface to enhance bioactivity and biocompatibility, thus increasing the potential of these nondegradable electrospun fiber scaffolds for abdominal wall replacement. Nevertheless, these results demonstrate the need for more studies to elucidate the mechanisms underlying cell/tissue-nanomaterial interactions in order to gain a better understanding of the risks involved in implantation of nanostructured biomaterials.

## Supporting Information

Figure S1 **Graphic illustration of the incisional hernia model.**
(PDF)

Figure S2 **Histological and morphometric details.**
(PDF)

Figure S3 **Example of omentum adhesion to a PET mesh.**
(PDF)
