# Supplementary material for: Foreign Body Reaction Associated with PET and PET/Chitosan Electrospun Nanofibrous Abdominal Meshes
Source: PLoS One. 2014 Apr 16;9(4):e95293. doi: 10.1371/journal.pone.0095293 (PMC3989343; doi:10.1371/journal.pone.0095293)

Figure S1. Graphic illustration of the incisional hernia model. (A) A 5 cm skin paramedian incision was made at the left size of abdomen using a sterile scalpel; (B) skin was dissected to expose the underlying abdominal fascia; (C,D) a1.5 x 1.5 cm defect of anterior abdominal wall was created by the complete resection of abdominal layers; (E) suture of mesh (2.0 x 2.0 cm) to the remaining muscle of the abdominal wall with interrupted suture and also with simple running suture all over the borders; (F) the skin was closed with intradermal suture.

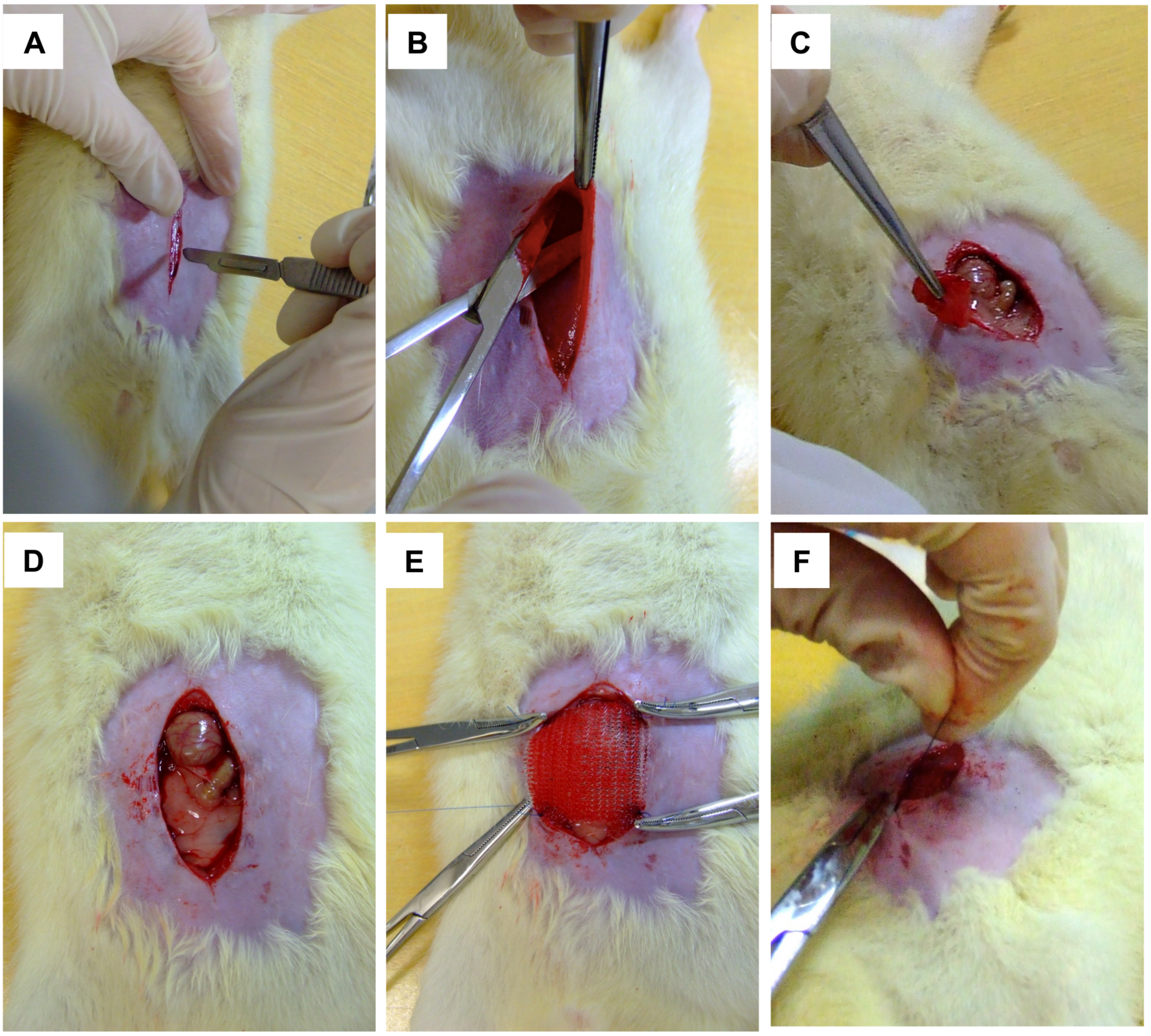

Supplement: Figure S1 — Graphic illustration of the incisional hernia model. (PDF) [file pone.0095293.s001.pdf]
