# Supplementary material for: Foreign Body Reaction Associated with PET and PET/Chitosan Electrospun Nanofibrous Abdominal Meshes
Source: PLoS One. 2014 Apr 16;9(4):e95293. doi: 10.1371/journal.pone.0095293 (PMC3989343; doi:10.1371/journal.pone.0095293)

Figure S2. Histological and morphometric details. (A) The animal tissue (abdominal mesh and surrounding tissue) was excised for histological analysis. (B) Tissue was transversally cut in two pieces, fixed in phosphate buffered formaldehyde solution (4%, pH 7.2, 0.1M), and embedded in paraffin. (C) Paraffin block was sectioned in a rotatory microtome (4 µm thickness). A single section at about 40 µm from the center of the excised tissue was selected. (D) The thickness of the foreign body granuloma (FBG) was calculated by subtracting the mesh thickness (z) from the total granuloma thickness (y) using 40x magnification micrographs and the ImageJ software – release 1.37c (n=8). For each section, the granuloma thickness was measured in 3 positions (m_1-first quarter, m_2- center, and m_3-third quarter).

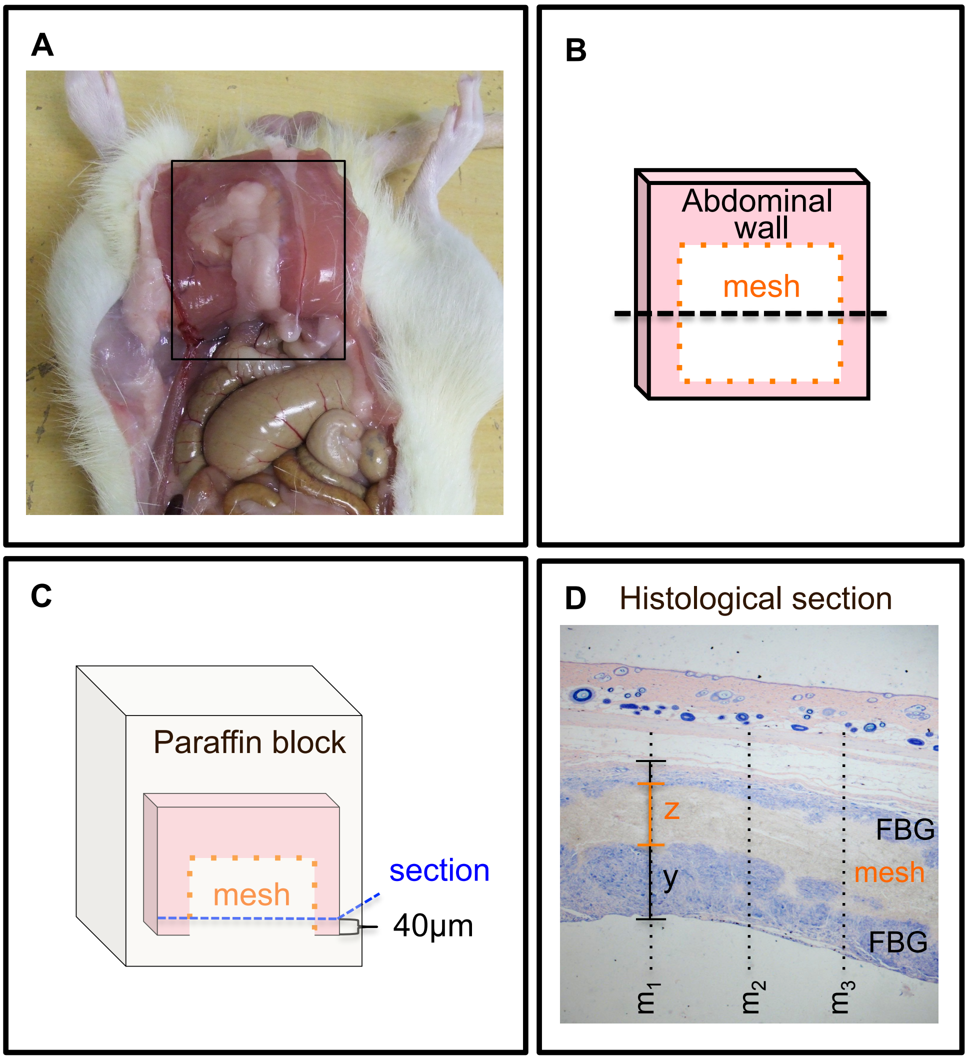

Supplement: Figure S2 — Histological and morphometric details. (PDF) [file pone.0095293.s002.pdf]
