# Supplementary figures and images for: Foreign Body Reaction Associated with PET and PET/Chitosan Electrospun Nanofibrous Abdominal Meshes
Source: PLoS One. 2014 Apr 16;9(4):e95293. doi: 10.1371/journal.pone.0095293 (PMC3989343; doi:10.1371/journal.pone.0095293)

**Figure S3. Example of omentum adhesion to a PET mesh.**

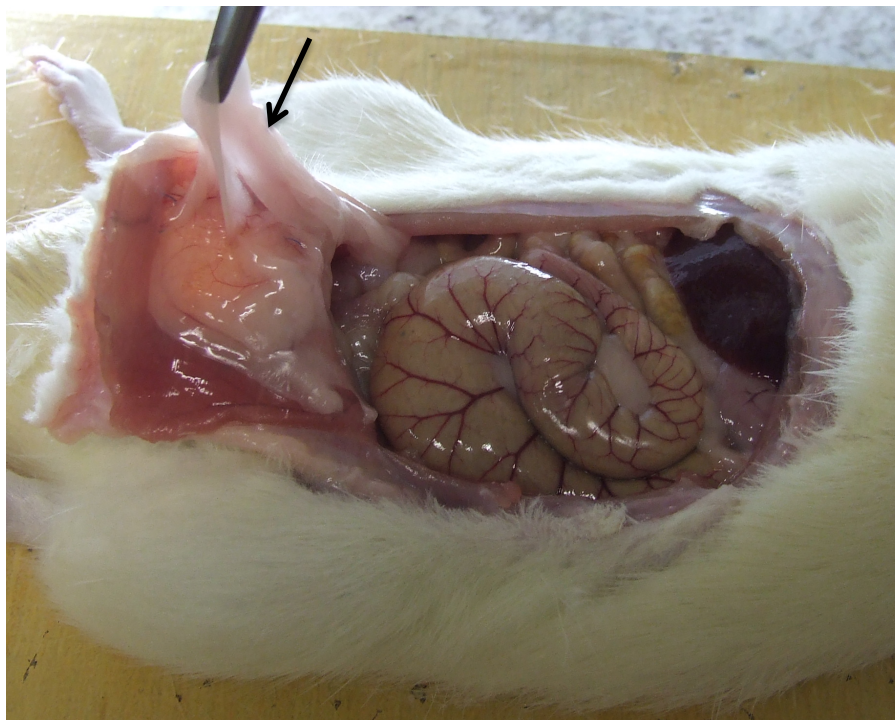

Supplement: Figure S3 — Example of omentum adhesion to a PET mesh. (PDF) [file pone.0095293.s003.pdf]
